# Infliximab‐induced seizures in a patient with Crohn’s disease: a case report

**DOI:** 10.1186/s12876-021-01780-y

**Published:** 2021-04-27

**Authors:** Zhijie Lv, Xiaoqi Zhang, Li Wu

**Affiliations:** 1 Department of Pharmacy, Sir Run Run Shaw Hospital, School of Medicine, Zhejiang University, 3 Qingchun Road, Hangzhou, 310006 Zhejiang China; 2 Department of gastroenterology, Nanjing Drum Tower Hospital, The Affiliated Hospital of Nanjing University Medical School, 321 Zhongshan Road, Nanjing, 210008 Jiangsu China; 3 Center of Clinical Evaluation, The First Affiliated Hospital of Zhejiang Chinese Medical University, 54 Youdian Road, Hangzhou, 310006 Zhejiang China

**Keywords:** Infliximab, Seizures, Crohn’s disease, Case report

## Abstract

**Background:**

Infliximab-induced seizures in patients with Crohn’s disease are extremely rare and the mechanism of infliximab-induced seizures is unclear.

**Case presentation:**

A 60-year-old woman with Crohn’s disease experienced infliximab-induced seizures, diagnosed on normal magnetic resonance imaging of the brain. Moreover, the rechallenge with infliximab was positive.

**Conclusions:**

Neurological assessment and tight clinical monitoring before and during therapy with infliximab should be performed in patients with pre-existing seizure disorders.

## Background

Infliximab is currently used as the first-line treatment for Crohn’s disease(CD). During the 20 years since its first approval in 1998, infliximab has revolutionized the treatment of inflammatory bowel disease(IBD). Over half a million patients have been treated with tumor necrosis factor (TNF)-α antagonists, but concerns regarding their safety have been raised worldwide [1].The most commonly reported adverse reactions to infliximab include acute or delayed hypersensitivity reactions; serious infections including reactivation of tuberculosis and hepatitis B virus; malignancy, especially lymphoma and hematologic reactions [2, 3]. However, new and rare side-effects have been increasingly reported in post-marketing reports. Here, we have reported a rare case of a patient with CD who experienced infliximab-induced seizures, diagnosed on normal magnetic resonance imaging (MRI) of the brain. Moreover, the rechallenge with infliximab was positive.

## Case presentation

A 60-year-old female presented to our hospital with a 10-day history of small intestinal stenosis due to CD. The patient was diagnosed with CD in 2015 due to chief complaints of abdominal pain and watery diarrhea (3−4 times per day). The patient’s medical history was unremarkable. She was treated with mesalazine (3 g/day), which partially alleviated the symptoms of abdominal pain and diarrhea (2−3 times per day). Ten days before admission, she underwent colonoscopy, but it was difficult to advance the colonoscope due to secondary intestinal stenosis. Biopsy and three-dimensional computed tomography of the small intestine confirmed the diagnosis of CD. Following admission to the hospital, a series of related examinations were performed. Electrocardiography revealed a normalized rhythm. Further evaluation revealed the following: slight leukopenia (leukocytes count, 3.2 × 10^9^/ L); serum albumin level, 36.1 g/L (normal range,40−55 g/L); platelet count, 123 × 10^9^/ L (normal range, 125−350 × 10^9^ /L); serum calcium level, 2.18 mmol/L (normal range, 2.25−2.75 mmol/L); fecal calprotectin level, 827.162 µg/g (normal range 0−50 µg/g) and serum magnesium level, 0.82 mmol/L (normal range, 0.7−1 mmol/L). T cell spot test for tuberculosis (T-SPOT.TB) revealed negative findings. She also had no history of alcohol use or drug abuse. Subsequently, treatment with infliximab was initiated at a dosage of 5 mg/kg. She did not experience any side effects after the first infliximab infusion. Two weeks later, she received the second infliximab infusion (5 mg/kg), but after 5 days, she suddenly developed short episodes of impairment of consciousness at home along with limbs twitches and the extroversion of eyeball. During the episode, her tongue was bitten, and her head was hurt. The episodes lasted for approximately 3 min, and she was taken to a local hospital for treatment by her family. However, she was not treated after observation at the hospital (details unspecified). According to the schedule, the patient received the third infliximab infusion at a loading dose of 5 mg/kg. She experienced repeated episodes after 5 days of the third infusion. She was taken to the local hospital, and craniocerebral CT showed no obvious abnormalities. She was then admitted to the Department of Neurology for further evaluation. Laboratory data showed normal findings, except a high L-cholesterol level (3.35 mmol/L; normal range, 1.89−3.1 mmol/L) and low lencocyte count (2.7 × 10^9^/L; normal range, 3.5−9.5 × 10^9^/L). All physical examination findings were unremarkable. On the third day of admission, she experienced another similar seizure episode. Therefore, diazepam (5 mg) and sodium valproate(800 mg) were administered intravenously to control the seizures. No recurrence was observed after treatment during hospitalization. She underwent a brain 3.0 T magnetic resonance angiography (MRA), which showed no apparent abnormality. Video electroencephalography revealed background activity in the alpha range with an amplitude reduction but a good waveform. On both sides of the forehead and temporal area, scattered sharp waves were observed; the waves were more obvious on the right side. During the monitoring period, the patient was cooperative and did not show any behavioural abnormalities. The Electroencephalogram (EEG) confirmed the diagnosis of seizures and so far we highly suspected that the occurrence of the seizures may be associated with the use of infliximab. The patient started maintenance therapy with valproic acid (500 mg/day) and was discharged after 6 days. Although there was a clear response to infliximab with a reduction of diarrhoea and abdominal pain, the infliximab treatment was ceased and no seizures occurred after discharge. Now thalidomide (25 mg/d) was used to maintain remission of CD. The patient was following up for the moment.

## Discussion and conclusions

Infliximab is a chimeric monoclonal antibody against the soluble and the membrane tumour necrosis factor (TNF)-α [4]. It is effective in inducing and maintaining remission in patients with moderate-to-severe CD refractory to conventional therapy [5]. However, administration of infliximab is associated with a well-recognized risk of infusion-related adverse events, such as infusion reactions, autoimmune disorders, malignancies, opportunistic infections, and serious infections [6]. The neurological effects of infliximab have also been reported. Headache is the most commonly reported, occurring in 12–18 % of patients studied in the clinical trial setting [7]. The other commonly reported events include peripheral neuropathy [8] and central nervous system and/or spinal cord demyelination. Most patients have good tolerance to infliximab; however, with its wide use in various autoinflammatory and immune diseases, it is expected that more adverse drug reactions will be reported in the future.

A literature review revealed that infliximab-related seizures have been rarely reported (Table 1). In 2008, a 14-year-old boy with active CD experienced probable occipital lobe seizures, followed by several episodes of generalized tonic clonic seizures, 5 days after the first infliximab administration [9]. In 2011, Francesco Brigo et al. [10] reported a case of a 74-year-old man with CD who developed a sudden seizures 2 days after the second infliximab administration. His medical history was notable for hepatitis C virus cirrhosis with normal liver function and for an ischemic right temporo-occipital stroke, but he did not have a history of previous seizures. Electroencephalography showed any paroxysmal activity. In 2011, Rosemary Haddock et al. [11] reported a case of posterior reversible encephalopathy syndrome in an 8-year-old girl with CD after infliximab administration and colectomy. In 2016, Chow et al. [12] reported a similar case of a 24-year-old woman who developed posterior reversible encephalopathy syndrome(PRES) after the second treatment with infliximab. Among the abovementioned, two cases occurred after the second injection of infliximab, and two occurred after the first injection of infliximab. There seemed to be no apparent consistency in the time of the occurrence of the adverse reaction and definitely none of the patients had a history of previous seizures.

**Table 1:** Summary of patient characteristics, seizures, and outcome

Patients	Age at presentation,gender	Inflammatory disorder	TNF-alpha inhibitor onset to seizures	Features of seizures	EEG	CSF	Other	Treatment for the seizures	Seizures outcome	Inflammatory disorder outcome	Study author and year of publication
1	14, male	Crohn’s disease	5 days after the first infliximab administration	Repeated episodes each lasting about 1 min and followed by several periods of generalized tonic clonic seizures lasting more than 6 min	Mild excess slow wave activity	Normal	MRI revealed abnormal T2 and fluid-attenuated inversion recovery signal hyperintensities in a broadly symmetrical distribution affecting the cerebellar hemispheres, occipital poles, medial parietal lobes, and peripheral frontal lobes	TNF-alpha inhibitor stopped, phenytoin	No more seizures occurred	Remission for 6 months and finally relapse, culminating in colectomy and ileostomy.	Zamvar,2009^[9]^
2	74, male	Crohn’s disease	2 days after the second infliximab administration	Impairment of consciousness, amnesia and arrest of volitional movements, confusion and disorientation, aggressiveness	Focal paroxysmal activity	Not recorded	MRI showed encephalopathy involving mainly cortical regions	TNF-alpha inhibitor stopped	No more seizures occurred	Not recorded	Brigo,2011^[10]^
3	24, female	Crohn’s disease	3 days following the second infliximab infusion	Experienced 2 episodes of generalized tonic clonic seizures	Diffuse nonspecific cerebral dysfunction	Not recorded	MRI showed scattered T2/FLAIR signal abnormalities in the sub-cortical white matter predominantly in the frontal and posterior parietal lobes	TNF-alpha inhibitor stopped	No more seizures occurred	Not recorded	Chow,2016^[12]^
4	8, female	Crohn’s disease	13 days after the first infliximab infusion	Nausea, visual disturbance, unresponsive dilated reactive pupils, bradycardic and hypertensive.	Right temporal lobe dysfunction	Normal	MRI showed abnormal high signal in the subcortical region, bilateral occipital lobes, and on the right side with extension to involve the right temporal region	TNF-alpha inhibitor stopped, benzodiazepine, phenytoin	Three focal seizures post discharge	Well controlled, and no further seizures at two year follow up.	Haddock,2011^[11]^

TNF, tumor necrosis factor; EEG, electroencephalogram; CSF, cerebrospinal fluid; MRI: magnetic resonance imaging

In our case, there was a direct correlation between seizures and infliximab administration. To the best of our knowledge, this is one of few case reports of infliximab-induced seizures. In contrast to the previous cases, our patient experienced the rechallenge events. Five days after the second infusion, the patient experienced actually a seizure, just failing to give enough attention. She again experienced seizures after the third infusion. This positive rechallenge was the strongest proof of side effects of infliximab. In the absence of infective, metabolic encephalopathy and other known etiologies, symptoms regressed quickly and completely. We also ruled out the possibility of seizures caused by other drugs because no special drugs were administered before the first three seizures except infliximab. MRA revealed no abnormalities. Based on the video electroencephalography findings, we speculated that the seizures were clearly associated with infliximab-related neurotoxicity. In previously reported cases, Posterior Reversible Encephalopathy Syndrome (PRES) has been reported, but in our case, both clinical symptoms and neuroradiological results were incompatible with the diagnosis of PRES. Therefore, this case was different from the other previously reported cases.

The mechanism of infliximab-induced seizures is unclear. However, it may be due to the systemic pro-inflammatory effects of α-TNF agents that cause an inflammatory response in the nerves [13]. Therefore, infliximab should be cautiously administered to patients to minimize possible morbidity for patients. Medication withdrawal is the first step in managing patients with suspected drug-induced neuropathy [14], The adverse events can occur in the initial stage of infliximab treatment during induction phase. Moreover, all cases reported thus date had no history of previous seizures and no other plausible cause of the seizures. Consequently,we must underline the possibility of serious and unexpected adverse reactions to infliximab, which are rare and unpredictable. Considering the elimination half-life of infliximab (10 days), we should pay particular attention to the adverse reactions after infliximab injection, especially before and after the second injection. Various neurological complications such as demyelination and peripheral neuropathy after treatment with TNF-α inhibitors have been reported [14, 15]. For patients with a history of demyelination, seizures or other serious neurological disorders, the use of TNF-α inhibitors may increase the risk of exacerbation of neurological symptoms. Neurological assessment and tight clinical monitoring before and during therapy with infliximab should be performed in patients with pre-existing seizure disorders. If absolutely necessary, prior assessment and appropriate measures should be still taken before initiating therapy. Further studies are still needed to evaluate the exact relationship between infliximab and seizures.

## Data Availability

This case report contains clinical data from the electronic medical record in the Nanjing Drum Tower Hospital. The datasets used during the current study are available from the corresponding author on reasonable request.

## Abbreviations

CD: Crohn’s disease
IBD: inflammatory bowel disease
TNF-α: Tumor necrosis factor-alpha
MRI: Magnetic resonance imaging
MRA: Magnetic Resonance Angiography
EEG: Electroencephalogram
CT: Computed tomography
PRES: Posterior Reversible Encephalopathy Syndrome
